# Reference Values for Cardiorespiratory Fitness in Healthy Koreans

**DOI:** 10.3390/jcm8122191

**Published:** 2019-12-12

**Authors:** Won Young Jang, Woohyeun Kim, Dong Oh Kang, Yoonjee Park, Jieun Lee, Jah Yeon Choi, Seung-Young Roh, Jin Oh Na, Cheol Ung Choi, Seung-Woon Rha, Chang Gyu Park, Hong Seog Seo, Soo Hyun Park, Saejong Park, Eung Ju Kim

**Affiliations:** 1 Cardiovascular Center, Korea University Guro Hospital, Seoul 08308, Korea; raph83@naver.com (W.Y.J.); coincidence1@naver.com (W.K.); gelly9@naver.com (D.O.K.); yoonjeedrpark@gmail.com (Y.P.); ch93lje@naver.com (J.L.); kekeruki@gmail.com (J.Y.C.); rsy008@gmail.com (S.-Y.R.); koolup93@gmail.com (J.O.N.); wmagpie@korea.ac.kr (C.U.C.); swrha617@yahoo.co.kr (S.-W.R.); parkcg@kumc.or.kr (C.G.P.); mdhsseo@unitel.co.kr (H.S.S.); 2 Korea Institute of Sport Science, Seoul 01794, Korea; otajulia@kspo.or.kr

**Keywords:** cardiorespiratory fitness, healthy volunteers, global health, ethnic groups, coronary artery disease

## Abstract

We investigated reference values for cardiorespiratory fitness (CRF) for healthy Koreans and Koreans with coronary heart disease (CHD) and used them to identify inter-ethnic differences in CRF, differences over time in CRF, and differences in CRF between the healthy population and patients with CHD. The study population for healthy Koreans was derived from the database of KISS FitS (Korea Institute of Sports Science Fitness Standards) between 2014 and 2015. The study population for Koreans with CHD was derived from the database of the Korea University Guro Hospital Cardiac Rehabilitation Registry between June 2015 and December 2018. In healthy Koreans, there was a significant difference between sex and age groups for VO_2_ max. The VO_2_ max of healthy Koreans differed from that of Westerners in age-related reference values. Our results were not significantly different from those of the Korean population in the past, except for a small decline in the young population. There seemed to be a clear inter-ethnic difference in CRF. We could also identify signs of small change in CRF in younger age groups. Therefore, CRF should be assessed according to ethnic or national standards, and it will be necessary to establish a reference for each nation or ethnicity with periodic updates.

## 1. Introduction

Cardiorespiratory fitness (CRF) is defined as the circulatory and respiratory ability to supply oxygen properly to skeletal muscles during physical activity. Many studies have shown that better CRF can lower the risk of cardiovascular disease (CVD) and all-cause mortality [1,2,3,4]. Recently, it has been argued that CRF should be regarded as one of the clinical vital signs, and it is expected that assessing CRF in clinical practice can improve patient management [5].

CRF can be measured directly by a conventional exercise test with gas analysis and can be estimated indirectly by exercise tests with various protocols [5]. Furthermore, non-exercise-based models that can predict CRF using clinical variables and can be easily assessed in clinical settings without an exercise test have been also reported [6,7,8,9]. Similar to these CRF measurements or estimates for individual subjects, it is important to establish a reference value that can be used to determine whether a subject is fit or unfit compared to a healthy population.

Since simple nomograms were introduced based on a U.S. cohort in the 1980s for men and the 1990s for women [10,11], there have been many efforts to update the reference for normal CRF. Recently, age-related mean reference values for American and Norwegian populations have been reported [12,13,14]. However, there has been no updated reference data for Asian populations. As CRF is influenced by genetic factors such as race [15,16,17], it is unreasonable to apply the reference values of Westerners to Asians. In addition, compared with the past, nutritional status has improved, but the obese population has increased significantly as the rate of sedentary life styles has increased. It could be also unreasonable to apply the past reference values to current subjects.

In this study, we investigated the CRF reference values for an Asian population using a Korean cohort from the 2010s and compared them with past Korean CRF results, as well as the reported CRF results for Westerners. We also compared the CRF of patients with coronary heart disease (CHD) with this reference value to determine the difference in CRF between patients with CHD and the healthy population.

## 2. Experimental Section

### 2.1. Study Population

This cross-sectional study consisted of two separate studies. One was a study to investigate the reference value for healthy adults, and the other was a study to measure the CRF in CHD patients. The study to investigate the reference value for healthy adults was conducted with healthy Koreans aged 19 years or older participating in the Korea Institute of Sports Science Fitness Standards (KISS FitS) project. KISS FitS was designed to measure the nationwide physical fitness of healthy Koreans, assess their health status, and suggest appropriate levels of fitness for disease prevention. We analyzed all subjects with available maximal oxygen uptake (VO_2_ max) who participated in KISS FitS between 2014 and 2015. The exclusion criteria for this project were pregnant women, patients with cardiovascular disease, renal disease, and systemic infections, and those with orthopedic injuries whose physical fitness could not be measured. This study was approved by the Korea Bioethics Committee for Institutional Bioethics (KISS-201504-EFS-002-01) and received voluntary consent from participants.

The study to measure the CRF in patients with CHD was conducted for those who underwent coronary angiography with or without revascularization and participated in cardiac rehabilitation at Korea University Guro Hospital between June 2015 and December 2018. Patients whose CRF could not be measured for the following reasons were excluded: (1) hemodynamic instability; (2) comorbidities such as pulmonary disease and/or orthopedic disease; and (3) non-cooperation due to a neurologic problem. This study was approved by the Institutional Review Board (IRB) of Korea University Guro Hospital. The requirement for written informed consent was waived because of the retrospective design of the study.

### 2.2. CRF of Healthy Koreans

Participants performed a treadmill exercise test with the Bruce protocol. The exercise test started at a speed of 1.7 mph and a slope of 10%, and the speed (2.5, 3.4, 4.2, 5.0 mph) and slope (12, 14, 16, 18, 20%) were increased every 3 min until the participants became exhausted. The criteria for termination of the exercise are as follows. (1) Rating of perceived exertion (RPE) of 17 or more; (2) the heart rate did not increase even when the intensity of exercise increased; (3) 85% of the heart rate reserve was reached; (4) Request for interruption by participants. VO_2_ max was estimated by converting the highest workload attained to exercise time [18].

### 2.3. CRF of Patients with CHD

CRF was measured as VO_2_ max directly through the gas exchange analyzer (Quark b2, COSMED, Rome, Italy) during the exercise test. The treadmill test was performed according to the modified Bruce protocol.

### 2.4. Statistical Analysis

Continuous data are presented as mean ± standard deviation (SD), whereas categorical data are presented as frequencies (percentages). The subjects were divided according to their ages at intervals of 10 years. CRF values were recorded and analyzed separately for men and women. The student’s *t*-test was used to assess the differences between the sexes. Analysis of variance (ANOVA) was used to evaluate the differences in VO_2_ max according to age groups. No formal statistical techniques were used when comparing cohorts between the West [13,14,19] and Korea, as well as past [10,11,20] and present [13,14,19] cohorts because of the unavailability of the individual participant data from the other cohorts. Considering that the VO_2_ max using the cycle ergometer was 10–20% lower than the VO_2_ max using the treadmill [21], VO_2_ max values of Danish [19] and past Korean cohorts [20] using the cycle ergometer were multiplied by 1.15 when comparing these VO_2_ max values. The CRF of patients with CHD was compared with the normal reference value using one-way ANOVA. Analyses were conducted using IBM SPSS Statistics 20.0 (Chicago, IL, USA).

## 3. Results

### 3.1. CRF of Healthy Koreans

A total of 2646 healthy subjects with available VO_2_ max data in the KISS FitS project were analyzed. Among them, 42.3% were men, and most metrics for men were higher than those for women except for age and percentage of body fat. The baseline characteristics are shown in Table 1. The mean VO_2_ max estimated from the treadmill test was presented for each age group by sex in Table 2. As the age increased, the VO_2_ max tended to decrease significantly. The VO_2_ max of men was significantly higher than that of women in all age groups, and the difference in VO_2_ max between the sexes decreased with age.

### 3.2. Predicted Exercise Capacity

**Figure 1:**
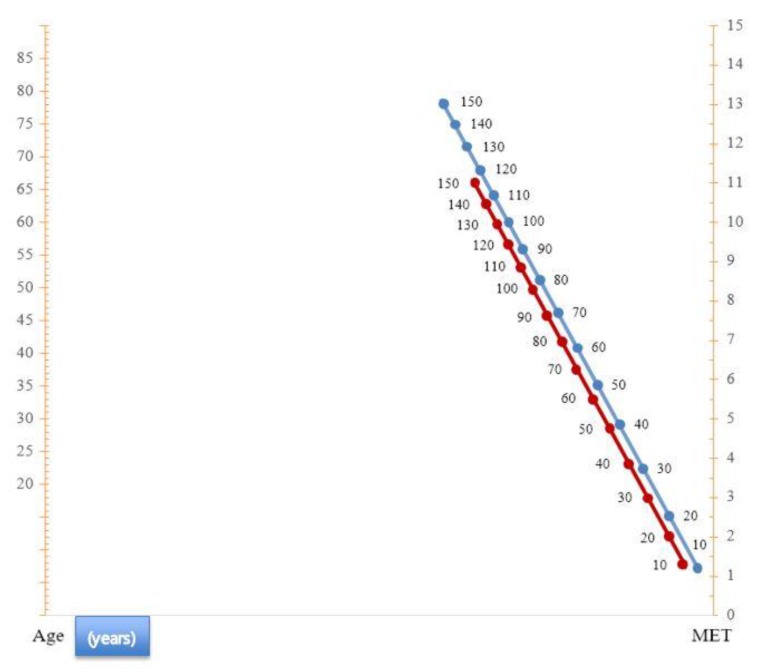
Nomogram of the percentage of predicted exercise capacity for age in healthy Korean participants. When a line connecting the subject’s age (on the left scale) and the metabolic equivalents (METS) value (on the right scale) is drawn, that line intersects the percentage line. The intersection point is the percentage of predicted exercise capacity for age. The red line is the women’s percentage line and the blue line is the male’s one.

The exercise capacity and age had a linear inverse correlation, and the following predictive equation was derived from a logistic linear regression: VO_2_ max = 50.54 − 0.26 × (age) for healthy Korean men; VO_2_ max = 40.0 − (0.22 × age) for healthy Korean women. A nomogram of healthy Korean CRF is shown in Figure 1.

### 3.3. Comparison of Koreans and Westerners: Inter-Ethnic Differences

**Figure 2:**
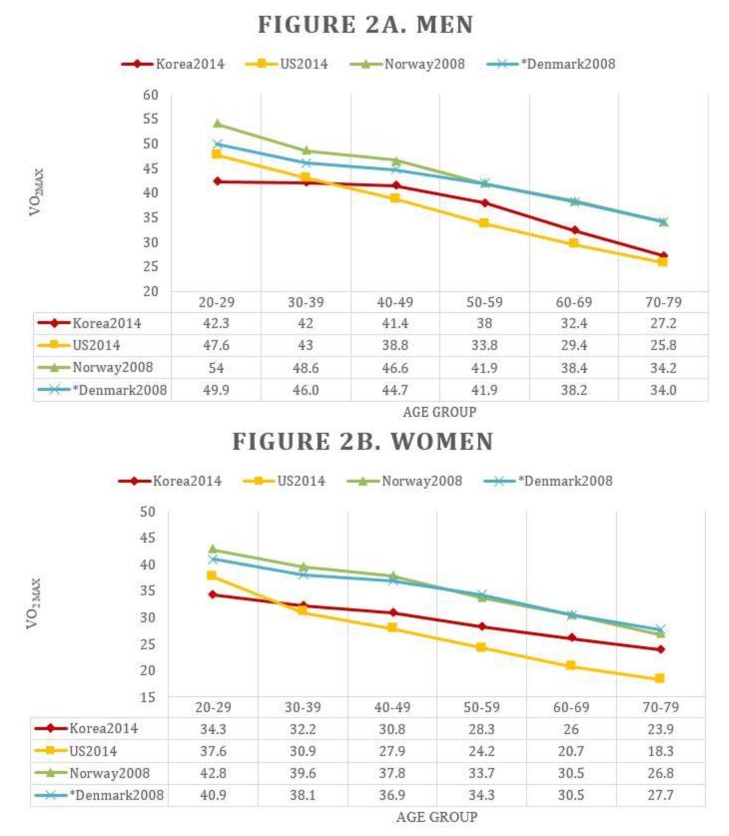
Comparison of cardiorespiratory fitness (CRF) of men (**A**) and women (**B**) between recent Korea, US, Norway, and Denmark. VO_2max_, maximal oxygen uptake (mL/kg/min). * CRF of Danish cohort was measured by cycle ergometer with the maximal power output (MPO) protocol. Due to the difference in VO_2max_ from exercise test methods, for simple comparison, the VO_2max_ measured by the cycle ergometer was multiplied by 1.15 times. The original VO_2max_ values of the Danish cohort can be found in Table S1.

In Figure 2 and Table S1, we compared the CRF of the previously reported American, Norwegian, and Danish cohorts to that of the Korean cohort [13,14,19]. Young healthy Korean men and women had a lower CRF compared than other cohorts. The annual decrease of CRF was the smallest in Korea, and therefore the difference in CRF between young and older participants was also the smallest in Korea. In particular, Korean women have lower CRF reduction with age, and those older than 30 years have a higher CRF than American women. As the age increased, the gap also increased. Similarly, the gap between Korean women and Scandinavian women decreased with age.

### 3.4. Comparison between Past and Present: Difference Over Time

**Figure 3:**
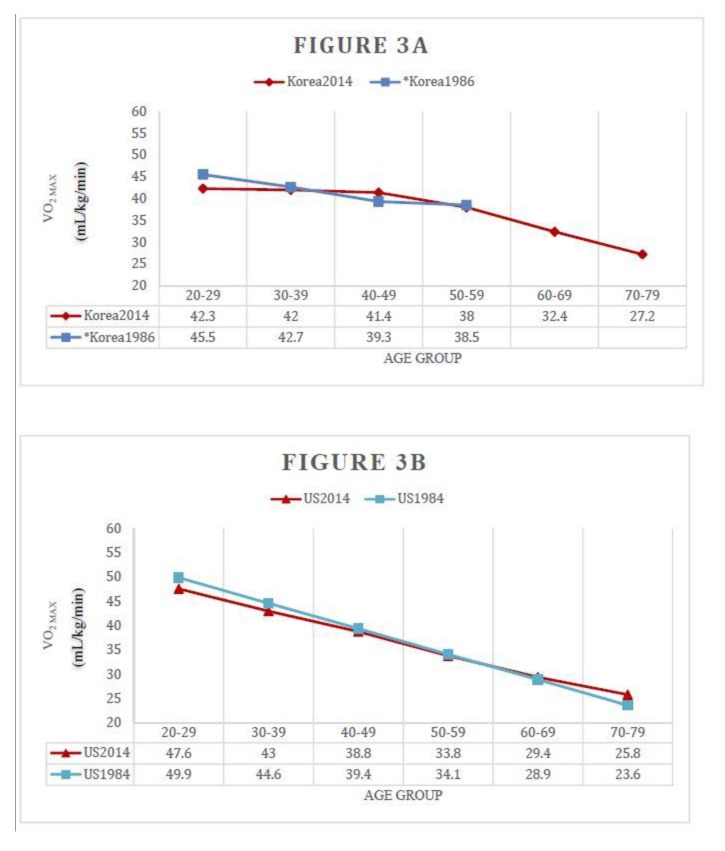

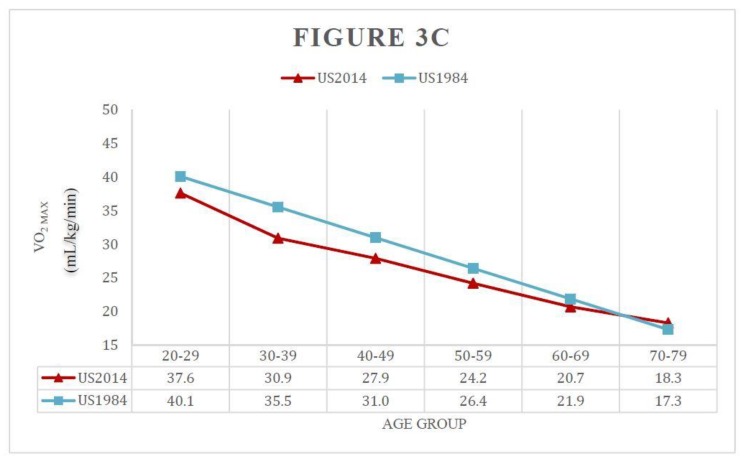
Comparison of cardiorespiratory fitness (CRF) between past and recent cohort. (**A**) Past and recent Korean men; (**B**) Past and recent US Men; (**C**) Past and recent US women. VO_2max_, maximal oxygen uptake (mL/kg/min). * CRF of past Korean participants was measured directly by cycle ergometer with gas analysis. Due to the difference in VO_2max_ from the exercise test methods, the VO_2max_ measured by cycle ergometer was multiplied by 1.15 for a more simple comparison. The original VO_2max_ values of past Korean participants can be found in Table S1.

Compared with previous Korean CRF data that were measured directly by a cycle ergometer with gas analysis [20], the VO_2_ max was different from our result (Table S1). Old Korean CRF values were lower than our result by around 1 metabolic equivalents (METs) in all age groups. However, considering the 10–20% difference between the cycle ergometer and the treadmill test, 21 CRF values were similar (Figure 3A). To confirm the changes in CRF over time, we compared the American cohort from the 1980s with those from the 2010s [10,14]. In the American cohort, there was also no significant difference in CRF over time (Figure 3B,C). Although only a numerical comparison was made without statistical techniques, the current values of CRF were consistently slightly lower in younger age groups than the past values in both the US and Korea (US men in 20s, Current *vs* past, 47.6 ± SD *vs* 49.9 ± SD; US women in 20s, Current *vs* past, 37.6 ± SD *vs* 40.1 ± SD; Korean men in 20s, Current *vs* past, 42.3 ± SD *vs* 45.5 ± SD).

### 3.5. CRF for Patients with CHD: Comparison with Reference Values of Healthy Koreans

From a total of 775 patients, 496 patients with available CRF data were included in this study. The population of patients with CHD consisted of 395 men (79.6%), with a mean age of 61.4 ± 10.1 years (Table 3). Furthermore, 82.5% of the patients had normal LV systolic function, and the average LV ejection fraction (EF) was 59.4%. Unstable angina was the most common diagnosis (42.1%). STEMI, NSTEMI, and stable angina accounted for 16.1%, 15.7%, and 26.0% of diagnoses, respectively. Baseline characteristics of CHD patients are presented in Table 3. In CHD patients, as in healthy adults, CRF decreased with increasing age, and the CRF of men was higher than that of women. In both men and women over all ages, the CRF of CHD patients was lower than that of the healthy population (Table 4). The difference in CRF between patients with CHD and the healthy population varied from 15–35% for each age group. When the nomogram was applied to patients with CHD, three-quarters of CHD patients (372, 75.0%) had a CRF of less than 85% of the predicted value, one of the indicators of a poor prognosis [11]. In particular, men with CHD aged 30–59 years have a significant reduction in CRF by more than 10 mL/kg/min. In addition, in this age group of men, more patients (152, 81.3%) had CRF less than 85% of the predicted value.

## 4. Discussion

There have been many studies that have presented mean reference values of CRF for each nation [22]. In a systemic review [22], Paap and Takken pointed out that the methodological quality of most studies was unsatisfactory when evaluated using the quality list based on the ATS/ACCP guidelines [23]. They noted that most of these studies had small sample sizes and used the cycle ergometer instead of the treadmill test. Only four studies [12,24,25,26] met the high quality criteria, and only two [12,24] of them used the treadmill test. Among these two studies, there were only 204 subjects in the study by Itoh et al. [24] and only one study had a relatively large sample size of 759 subjects who performed treadmill tests [12]. Recently, well-designed studies that measured CRF directly using the treadmill test have been published for relatively large populations, especially for Westerners [12,13,14]. Although there have been attempts to present age-related mean reference values for Asian populations [24,27,28,29], these studies also had small sample sizes and/or used cycle ergometers instead of the treadmill test.

As far as we know, this is the first study to investigate the reference values of CRF using the treadmill test for Asians with a large sample size and high quality. Although the study was confined to Koreans, it was meaningful because it was aimed at a relatively large population group as compared with the previous studies of Asian populations [24,27,28,29]. Another advantage of this study was that the percentage of predicted exercise capacity of Koreans by sex and age can be easily ascertained through the nomogram. Similar to other studies conducted in different countries [10,11,13,14,19], the following was also found in Korean CRF: (1) as age increases, CRF decreases, (2) CRF was higher in men than in women of all ages, and (3) the decrease in CRF according to age was greater for men than for women, and the difference in CRF between men and women decreased with age.

In this study, we also objectively showed the difference of CRF between an Eastern and Western population. The age-specific reference of CRFs were reported differently in each study [10,11,13,14,19], with Scandinavian people having the highest CRF over all ages (Figure 2, Table S1 in the supplement). The Danish study is a cycle-based study, which was difficult to compare directly, but considering that CRF measurements using cycles were 10–20% lower than those measured using the treadmill [21], the age-specific reference of CRF in Norway and that in Denmark were similar. On the other hand, there were differences between Korean, Americans, and Scandinavians. In Korean populations, CRF reduction by age was characteristically smaller than other Westerners. As a result, the CRF of Americans was higher than that of Koreans in the younger age group, but the CRF of Koreans was higher than that of Americans in the age groups of 30–40 years and older. In particular, Korean women had the least decrease in CRF according to age, and older women were closer to Scandinavian CRF.

In addition, this study provided clues to indirectly compare the past and present CRF. We compared the results of this study with those of a past Korean study [20] (Figure 3A and Table S1). A simple numerical comparison suggests that the CRF of Koreans has improved over the years for all ages; however, this finding requires further interpretation (Table S1). The past study with Koreans was conducted using a cycle ergometer with only 82 men [20]. Subsequently, there might be limitations in the comparison with current data. Moreover, as mentioned above, considering that VO_2_ max is approximately 10–20% lower when using a cycle ergometer compared to a treadmill test [21], our results can be judged to be similar when compared with the past (Figure 3a). Intriguingly, the age-related mean reference values of Americans in the 1980s and 2010s also did not change much [10,14] (Figure 3B,C). Despite medical progress and lifestyle changes, this finding that CRF has remained almost unchanged for 30 years might indicate that CRF is affected more by genetic factors.

There were minor but interesting changes, which might be a coincidence, but in both the U.S. and Korea, the current CRF was consistently slightly lower in the younger age groups compared to the past (Figure 3). Intriguingly, in a study of 18-year-old men in Norway, Dyrstad et al. reported that CRF in young men had decreased over the 20 years [30]. Because it was accompanied by an increase in body weight and BMI over the same period, they argued that a decrease in CRF in younger men might have been due to a decrease in daily physical activity. Considering the increasing trend of obesity worldwide, including Korea [31,32,33,34], the observed decrease in CRF in younger age groups in the U.S. and Korea is convincing. Because the decrease of CRF in younger age groups may indicate a decrease of CRF in the whole population of the future, a periodic assessment of CRF is needed along with lifestyle modifications.

Another advantage of this study was that the CRF of patients with CHD was compared with that of a healthy population. Considering previous studies where CRF had a strong inverse correlation with incident CHD [35] and a measured CRF of less than 85% of the predicted value suggested a poor prognosis in the general population [11], patients with CHD may have a lower CRF than the healthy population. In our study, the decrease in CRF of patients with CHD was objectively confirmed, with most patients having CRF <85% of the predicted values.

This study has some limitations. Unlike previous studies in which CRF was directly measured using gas analysis during the treadmill test [12,13,14,20], CRF was indirectly estimated from the treadmill test in this study for a healthy Korean reference. Although the measured and estimated CRF were moderately correlated, there was a clear difference between the two values [36]. Because this was a retrospective cross-sectional study based on a cohort that has already been investigated, one limitation of our study was that CRF was estimated indirectly. However, at least for Koreans, the estimated CRF of the healthy population by the treadmill test with the Bruce protocol was relatively accurate, with an error rate of less than 1.5%, compared to the measured CRF [37]. Thus, although we used the estimated CRF instead of the directly measured CRF in this study, it was not unreasonable to compare the estimated CRF of our study with the directly measured CRF of other studies.

When comparing the CRF between Korean and Westerners, or comparing the past and current CRF, we simply compared the results observed in each study without statistical techniques. Therefore, it cannot be interpreted that the inter-ethnic difference of CRF has a statistical meaning, but it should be satisfied only to confirm tendency.

Because it has not been yet proven in Korea as to whether CRF values using the treadmill test and cycle ergometer differ by 10–20%, comparing the past and current CRF of Koreans can be criticized as being logically outrageous. In this regard, it may be also necessary to investigate the CRF reference values using a cycle ergometer as well as treadmill tests for Koreans.

## 5. Conclusions

In our study, the CRF of healthy Koreans differed from that of Westerners in age-related reference values. There seemed to be a clear inter-ethnic difference in CRF. Therefore, CRF should be assessed according to ethnic or national standards, and it will be necessary to establish a reference for each nation or ethnicity. While it looked like there was no significant change in CRF over time in the same ethnic group, we could also identify signs of small change in CRF in younger age groups. It will be necessary to periodically update these references. We also found that the CRF of patients with CHD was significantly lower than that of the healthy population. Further studies are needed to determine the effect of decreased CRF in patients with CHD.

## Figures and Tables

**Table 1:** Baseline characteristics of the healthy Korean participants.

	Men	Women	*p*-Value
Sample size, number	1119 (42.3)	1527 (57.7)	
Age, years	46.4 (16.1)	52.8 (14.9)	<0.001
Height, cm	170.8 (6.6)	157.0 (6.0)	<0.001
Weight, kg	72.2 (10.9)	58.2 (8.3)	<0.001
BMI, kg/m^2^	24.7 (3.0)	23.6 (3.1)	<0.001
WC, cm	86.6 (7.7)	83.0 (8.5)	<0.001
Resting SBP, mmHg	126.0 (12.1)	119.1 (14.5)	<0.001
Resting DBP, mmHg	79.4 (10.8)	74.1 (9.0)	<0.001
Percent body fat, %	22.8 (6.0)	32.2 (6.4)	<0.001
Lean mass, kg	31.1 (4.4)	21.0 (2.7)	<0.001
Smoking, number	233(20.8)	9(0.6)	<0.001
Hypertension, number	220(19.7)	325(21.3)	0.340
Dyslipidemia, number	135(12.1)	241(15.8)	0.009
Family history *, number	55(4.9)	78(5.1)	0.853

Data are presented as mean (standard deviation), or as numbers followed by percentages. * Family history of cardiovascular disease. BMI, body mass index; WC, waist circumference; SBP, systolic blood pressure; DBP, diastolic blood pressure.

**Table 2:** Cardiorespiratory fitness of the healthy Korean participants.

Men	VO_2max_ (mL/kg/min)			Women	VO_2max_ (mL/kg/min)		
Age	Mean ± SD	N	*p*-Value for Trend *	Age	Mean ± (SD)	N	*p*-Value for Trend *
19–29	42.3 ± 6.3	209		19–29	34.3 ± 4.3	110	
30–39	42.0 ± 5.0	170		30-39	32.2 ± 4.5	211	
40–49	41.4 ± 5.6	238		40–49	30.8 ± 4.6	284	
50–59	38.0 ± 5.7	274	<0.01	50–59	28.3 ± 4.6	367	<0.01
60–69	32.4 ± 6.2	134		60–69	26.0 ± 5.7	336	
70–79	27.2 ± 5.6	83		70–79	23.9 ± 4.4	195	
>80	24.1 ± 4.0	11		>80	21.0 ± 3.7	24	
Total	38.6 ± 7.4	1119		total	28.5 ± 5.8	1527	

Data are presented as mean ± standard deviation. Analysis of variance was used to evaluate the differences in VO_2max_ according to age. * *p*-value for the trend refers to testing for the trend of VO_2max_ by decades of age by ANOVA. SD, standard deviation; VO_2max_, maximal oxygen uptake; N, number.

**Table 3:** Baseline characteristics of the Korean patients with coronary heart disease.

	Men	Women	*p*-Value
Sample size, number	395 (79.6)	101 (20.4)	
Age, year	60.4 ± 10.1	65.7 ± 8.6	<0.001
Height, cm	172.4 ± 75.7	153.4 ± 6.1	<0.001
Weight, kg	71.9 ± 12.3	60.4 ± 9.7	<0.001
BMI, kg/m^2^	25.2 ± 3.9	25.4 ± 3.2	0.675
Waist-to-hip ratio	0.9 ± 0.1	0.9 ± 0.1	0.687
Basal metabolic rate	1527.4 ± 177.3	1207.1 ± 126.5	<0.001
Systolic Blood Pressure	122.9 ± 14.9	123.4 ± 16.3	0.769
Diastolic Blood Pressure	73.9 ± 11.4	71.9 ± 11.7	0.119
LVEF	59.0 ± 8.2	60.0 ± 7.8	0.036
>50%	321 (81.3)	88 (87.1)	0.167
≤50%	28 (7.1)	6 (5.9)	0.684
≤45%	22 (5.6)	3(3.0)	0.287
≤35%	10 (2.5)	2(2.0)	0.748
Underlying disease			
Hypertension	274 (69.4)	78 (77.2)	0.120
Diabetes mellitus	155 (39.2)	34 (33.7)	0.303
Hyperlipidemia	208 (52.7)	59 (58.4)	0.300
Chronic renal insufficiency	28 (7.1)	9 (8.9)	0.534
Diagnosis			
STEMI	71 (18.0)	9 (8.9)	0.027
NSTEMI	65 (16.5)	13 (12.9)	0.377
Unstable angina	157 (39.7)	52 (51.5)	0.033
Stable angina	102 (25.8)	27 (26.7)	0.852

Data are presented as mean (standard deviation), or as numbers followed by percentages. LVEF, left ventricular ejection fraction; STEMI, ST-elevation myocardial infarction; NSTEMI, non-ST-elevation myocardial infarction; VO_2max_, maximal oxygen uptake; METs, metabolic equivalents.

**Table 4:** Comparison of cardiorespiratory fitness between healthy population and patients with coronary heart disease.

	Men					Women				
	Healthy		CHD			Healthy		CHD		
Age	VO_2max_ (mL/kg/min) Mean ± SD	N	VO_2max_ (mL/kg/min) Mean ± SD	N	*p*-value	VO_2max_ (mL/kg/min) Mean ± SD	N	VO_2max_ (mL/kg/min) Mean ± SD	N	*p*-value
19–29	42.3 ± 6.3	209				34.3 ± 4.3	110			
30–39	42.0 ± 5.0	170	28.6 ± 5.0	7	<0.001	32.2 ± 4.5	211			0.014
40–49	41.4 ± 5.6	238	28.3 ± 5.5	52	<0.001	30.8 ± 4.6	284	24.2 ± 3.0	3	<0.001
50–59	38.0 ± 5.7	274	25.9 ± 5.2	128	<0.001	28.3 ± 4.6	367	23.1 ± 4.1	19	<0.001
60–69	32.4 ± 6.2	134	25.5 ± 5.2	125	<0.001	26.0 ± 5.7	336	22.3 ± 3.8	46	<0.001
70–79	27.2 ± 5.6	83	21.9± 5.4	73	<0.001	23.9 ± 4.4	195	18.0± 3.9	29	<0.001
>80	24.1 ± 4.0	11	20.0 ± 5.5	10	0.068	21.0 ± 3.7	24	13.5 ± 3.2	4	0.001

Data are presented as mean ± standard deviation. Independent-sample t-test was used to compare CRF in healthy population and CRF in CHD patients. CHD, coronary heart disease; SD, standard deviation; VO_2max_, maximal oxygen uptake; N, number.

## Supplementary Materials

The following are available online at https://www.mdpi.com/2077-0383/8/12/2191/s1, Table S1: Comparison with previous studies to evaluate a reference cardiorespiratory fitness of healthy population.

## References

[1] Kodama S., Saito K., Tanaka S., Maki M., Yachi Y., Asumi M., Sugawara A., Totsuka K., Shimano H., Ohashi Y. (2009). Cardiorespiratory fitness as a quantitative predictor of all-cause mortality and cardiovascular events in healthy men and women: A meta-analysis. JAMA.

[2] Blair S.N., Kohl H.W., Paffenbarger R.S., Clark D.G., Cooper K.H., Gibbons L.W. (1989). Physical fitness and all-cause mortality. A prospective study of healthy men and women. JAMA.

[3] Gulati M., Pandey D.K., Arnsdorf M.F., Lauderdale D.S., Thisted R.A., Wicklund R.H., Al-Hani A.J., Black H.R. (2003). Exercise capacity and the risk of death in women: The St James Women Take Heart Project. Circulation.

[4] Myers J., Prakash M., Froelicher V., Do D., Partington S., Atwood J.E. (2002). Exercise capacity and mortality among men referred for exercise testing. N. Engl. J. Med..

[5] Ross R., Blair S.N., Arena R., Church T.S., Despres J.P., Franklin B.A., Haskell W.L., Kaminsky L.A., Levine B.D., Lavie C.J. (2016). Importance of Assessing Cardiorespiratory Fitness in Clinical Practice: A Case for Fitness as a Clinical Vital Sign: A Scientific Statement from the American Heart Association. Circulation.

[6] Jurca R., Jackson A.S., La Monte M.J., Morrow J.R., Blair S.N., Wareham N.J., Haskell W.L., van Mechelen W., Church T.S., Jakicic J.M. (2005). Assessing cardiorespiratory fitness without performing exercise testing. Am. J. Prev. Med..

[7] Cao Z.B., Miyatake N., Higuchi M., Miyachi M., Ishikawa-Takata K., Tabata I. (2010). Predicting VO2max with an objectively measured physical activity in Japanese women. Med. Sci. Sports Exerc..

[8] Cao Z.B., Miyatake N., Higuchi M., Miyachi M., Tabata I. (2010). Predicting VO (2max) with an objectively measured physical activity in Japanese men. Eur. J. Appl. Physiol..

[9] Mailey E.L., White S.M., Wojcicki T.R., Szabo A.N., Kramer A.F., McAuley E. (2010). Construct validation of a non-exercise measure of cardiorespiratory fitness in older adults. BMC Public Health.

[10] Morris C.K., Myers J., Froelicher V.F., Kawaguchi T., Ueshima K., Hideg A. (1993). Nomogram based on metabolic equivalents and age for assessing aerobic exercise capacity in men. J. Am. Coll. Cardiol..

[11] Gulati M., Black H.R., Shaw L.J., Arnsdorf M.F., Merz C.N., Lauer M.S., Marwick T.H., Pandey D.K., Wicklund R.H., Thisted R.A. (2005). The prognostic value of a nomogram for exercise capacity in women. N. Engl. J. Med..

[12] Edvardsen E., Hansen B.H., Holme I.M., Dyrstad S.M., Anderssen S.A. (2013). Reference values for cardiorespiratory response and fitness on the treadmill in a 20- to 85-years-old population. Chest.

[13] Loe H., Rognmo O., Saltin B., Wisloff U. (2013). Aerobic capacity reference data in 3816 healthy men and women 20–90 years. PLoS ONE.

[14] Kaminsky L.A., Arena R., Myers J. (2015). Reference Standards for Cardiorespiratory Fitness Measured with Cardiopulmonary Exercise Testing: Data from the Fitness Registry and the Importance of Exercise National Database. Mayo Clin. Proc..

[15] Bouchard C., Dionne F.T., Simoneau J.-A., Boulay M.R. (1992). 2: Genetics of aerobic and anaerobic performances. Exerc. Sport Sci. Rev..

[16] Sanders L.F., Duncan G.E. (2006). Population-based reference standards for cardiovascular fitness among U.S. adults: NHANES 1999–2000 and 2001–2002. Med. Sci. Sports Exerc..

[17] Wang C.Y., Haskell W.L., Farrell S.W., Lamonte M.J., Blair S.N., Curtin L.R., Hughes J.P., Burt V.L. (2010). Cardiorespiratory fitness levels among US adults 20–49 years of age: Findings from the 1999–2004 National Health and Nutrition Examination Survey. Am. J. Epidemiol..

[18] Bruce R.A., Kusumi F., Hosmer D. (1973). Maximal oxygen intake and nomographic assessment of functional aerobic impairment in cardiovascular disease. Am. Heart J..

[19] Eriksen L., Gronbaek M., Helge J.W., Tolstrup J.S. (2016). Cardiorespiratory fitness in 16,025 adults aged 18–91 years and associations with physical activity and sitting time. Scand. J. Med. Sci. Sports.

[20] Shim W.J., Suh S.K. (1986). The Stress Exercise Test and Oxygen Uptake in Normal Korean Men. Korean J. Intern. Med..

[21] Arena R., Myers J., Guazzi M. (2010). The future of aerobic exercise testing in clinical practice: Is it the ultimate vital sign?. Future Cardiol..

[22] Paap D., Takken T. (2014). Reference values for cardiopulmonary exercise testing in healthy adults: A systematic review. Expert Rev. Cardiovasc. Ther..

[23] Society A.T. (2003). ATS/ACCP statement on cardiopulmonary exercise testing. Am. J. Respir. Crit. Care Med..

[24] Itoh H., Ajisaka R., Koike A., Makita S., Omiya K., Kato Y., Adachi H., Nagayama M., Maeda T., Tajima A. (2013). Heart rate and blood pressure response to ramp exercise and exercise capacity in relation to age, gender, and mode of exercise in a healthy population. J. Cardiol..

[25] Davis J.A., Storer T.W., Caiozzo V.J. (1997). Prediction of normal values for lactate threshold estimated by gas exchange in men and women. Eur. J. Appl. Physiol. Occup. Physiol..

[26] Storer T.W., Davis J.A., Caiozzo V.J. (1990). Accurate prediction of VO2max in cycle ergometry. Med. Sci. Sports Exerc..

[27] Singh R., Singh H.J., Sirisinghe R.G. (1989). Cardiopulmonary fitness in a sample of Malaysian population. Jpn. J. Physiol..

[28] Ong K.C., Loo C.M., Ong Y.Y., Chan S.P., Earnest A., Saw S.M. (2002). Predictive Values for Cardiopulmonary Exercise Testing in Sedentary Chinese Adults, Respirology.

[29] John N., Thangakunam B., Devasahayam A.J., Peravali V., Christopher D.J. (2011). Maximal oxygen uptake is lower for a healthy Indian population compared to white populations. J. Cardiopulm. Rehabil. Prev..

[30] Dyrstad S.M., Aandstad A., Hallen J. (2005). Aerobic fitness in young Norwegian men: A comparison between 1980 and 2002. Scand. J. Med. Sci. Sports.

[31] Wang Y.C., McPherson K., Marsh T., Gortmaker S.L., Brown M. (2011). Health and economic burden of the projected obesity trends in the USA and the UK. Lancet (Lond. UK).

[32] Finucane M.M., Stevens G.A., Cowan M.J., Danaei G., Lin J.K., Paciorek C.J., Singh G.M., Gutierrez H.R., Lu Y., Bahalim A.N. (2011). National, regional, and global trends in body-mass index since 1980: Systematic analysis of health examination surveys and epidemiological studies with 960 country-years and 9.1 million participants. Lancet (Lond. UK).

[33] Khang Y.-H., Yun S.-C. (2010). Trends in general and abdominal obesity among Korean adults: Findings from 1998, 2001, 2005, and 2007 Korea National Health and Nutrition Examination Surveys. J. Korean Med. Sci..

[34] Ogden C.L., Carroll M.D., Curtin L.R., McDowell M.A., Tabak C.J., Flegal K.M. (2006). Prevalence of overweight and obesity in the United States, 1999–2004. JAMA.

[35] Letnes J.M., Dalen H., Vesterbekkmo E.K., Wisloff U., Nes B.M. (2018). Peak oxygen uptake and incident coronary heart disease in a healthy population: The HUNT Fitness Study. Eur. Heart J..

[36] Loprinzi P.D., Edwards M.K., Addoh O., Bentley J.P. (2018). Evaluation of the convergent validity of an estimated cardiorespiratory fitness algorithm. Eur. J. Appl. Physiol..

[37] Park S., Lee M., Ahn H. (2014). Validation of prediction equations for VO2max using bruce protocol. Korean J. Meas. Eval. Phys. Educ. Sport Sci..

