# Supplementary material for: Reference Values for Cardiorespiratory Fitness in Healthy Koreans
Source: J Clin Med. 2019 Dec 12;8(12):2191. doi: 10.3390/jcm8122191 (PMC6947150; doi:10.3390/jcm8122191)
Supplement: Supplementary file 1 [file jcm-08-02191-s001.pdf]

Table S1. Comparison with previous studies to evaluate a reference cardiorespiratory fitness of healthy population.

	**Study**		**Shim et al. [20]**	**Morris et al. [10]**	**Kaminsky et al. [14]**	**Loe et al. [13]**	**Eriksen et al. [19]**
**Male**	**Country**	**Korea**	**Korea**	**USA**	**USA**	**Norway**	**Denmark**
	**Recruitment date**	**2014–2015**	**1986**	**1984–1989**	**2014**	**2006–2008**	**2007–2008**
	**Methods**	**Treadmill**	**Cycle**	**Treadmill**	**Treadmill**	**Treadmill**	**Cycle**
	**Assessment**	**Indirect**	**Direct**	**Direct**	**Direct**	**Direct**	**Direct**
	20–29	42.3	39.6	49.9	47.6	54	43.4
**VO_{2max}**	30–39	42.0	37.1	44.6	43	48.6	40
(mL/kg/min)	40–49	41.4	34.2	39.4	38.8	46.6	38.9
	50–59	38.0	33.5	34.1	33.8	41.9	36.4
	60–69	32.4		28.9	29.4	38.4	33.2
	70–79	27.2		23.6	25.8	34.2	29.6
	**Study**			**Gulati et al. [11]**	**Kaminsky et al.**	**Loe et al.**	**Eriksen et al.**
**Female**	**Country**	**Korea**		**USA**	**USA**	**Norway**	**Denmark**
	**Recruitment date**	**2014–2015**		**1990–1995**	**2014**	**2006–2008**	**2007–2008**
	**Methods**	**Treadmill**		**Treadmill**	**Treadmill**	**Treadmill**	**Cycle**
	**Assessment**	**Indirect**		**Direct**	**Direct**	**Direct**	**Direct**
	20–29	34.3		40.1	37.6	42.8	35.6
**VO_{2max}**	30–39	32.2		35.5	30.9	39.6	33.1
(mL/kg/min)	40–49	30.8		31.0	27.9	37.8	32.1
	50–59	28.3		26.4	24.2	33.7	29.8
	60–69	26.0		21.9	20.7	30.5	26.5
	70–79	23.9		17.3	18.3	26.8	24.1

VO_{2max}, Maximal oxygen uptake
